# Case Marking in Hindi as the Weaker Language

**DOI:** 10.3389/fpsyg.2019.00461

**Published:** 2019-03-19

**Authors:** Silvina Montrul, Archna Bhatia, Rakesh Bhatt, Vandana Puri

**Affiliations:** ^1^Department of Spanish and Portuguese/Department of Linguistics, University of Illinois at Urbana-Champaign, Urbana, IL, United States; ^2^Florida Institute for Human and Machine Cognition, Pensacola, FL, United States

**Keywords:** Hindi, dominance, heritage speakers, second language, case, ergativity, differential object marking

## Abstract

Does language dominance modulate knowledge of case marking in Hindi-speaking bilinguals? Hindi is a split ergative language with a rich morphological case system. Subjects of transitive perfective predicates are marked with ergative case (-*ne*). Human specific direct objects, indirect objects, and dative subjects are marked with the particle -*ko*. We compared knowledge of case marking in Hindi–English bilinguals with different dominance patterns: 23 balanced bilinguals and two groups of bilinguals with Hindi as their weaker language: 24 L2 learners of Hindi with age of acquisition (AoA) of Hindi in adulthood and 26 Hindi heritage speakers with AoA of Hindi since birth in oral production and acceptability judgments. The balanced bilinguals outperformed the English-dominant bilinguals; the L2 learners and the heritage speakers, who showed similar lower command of the Hindi case marking system, with the exception of -*ko* marking as a function of specificity with direct objects. We consider how dominant language transfer, AoA of Hindi, and input factors may explain the acquisition and knowledge of morphology in Hindi as the weaker language.

## Introduction

Bilinguals know two or more languages and may use them to different degrees. Although the term *bilingual* continues to conjure stable and equally highly proficient linguistic knowledge and use of two languages, the reality is that most bilinguals have unequal command of the two languages overall, by language skills, and in specific linguistic domains. Dominance is the relative weight and relationship of the two languages of a bilingual in terms of language use and degree of proficiency (Silva-Corvalán and Traffers-Dallers, 2016), with the two languages having relatively similar strength, or one being stronger/weaker than the other. Factors that contribute to language dominance may include age of acquisition (AoA) or age of bilingualism, estimations of language input, degree of language use, and proficiency in each language (Montrul, 2016a). Bilingual balance or imbalance may be a reflection of the Complementarity Principle (Grosjean, 2008): the idea that bilinguals use their languages in different situations and for different purposes along the lifespan.

**FIGURE 1:**
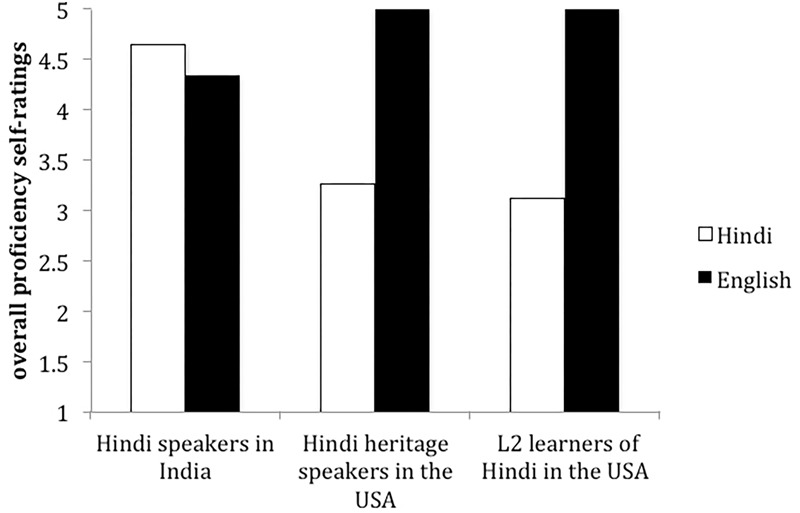
Dominance profiles of the bilinguals tested.

Does language dominance modulate knowledge of specific structural properties of a language in bilinguals? How does AoA and context of learning affect the acquisition of a weaker language? Does acquisition of a language very early in a naturalistic setting always have a long-term advantage? We answer these questions by looking at the linguistic situation depicted in Figure 1: we compare the linguistic abilities of Hindi–English bilinguals with different patterns of dominance (balanced vs. unbalanced bilinguals), and within the unbalanced groups we include bilinguals who share the same dominance pattern–English is the dominant language and Hindi is the weaker language–but differ in their AoA of their weaker language and in the context of learning: heritage speakers of Hindi and second language (L2) learners of Hindi in the United States.

For L2 learners the weaker language is the L2 but for heritage speakers it is the L1 [or language A (LA) if acquired simultaneously with language B (LB)]. L2 learners are late bilinguals because the L2 is typically acquired around or after puberty and in an instructed setting. In the case of heritage speakers–early bilinguals–their L1 is a minority language and it may become weak over time due to shift in childhood, becoming secondary in several domains of use. The majority language ends up being the dominant language in early adulthood and, if the heritage language is learned simultaneously with the majority language from birth, the heritage language can lag in development during the school-age period (Polinsky, 2006; Rothman, 2009; Carreira and Kagan, 2011; Montrul and Ionin, 2012; Montrul, 2016b). Yet, the weaker language in heritage speakers is considered a native language (Montrul, 2013; Rothman and Treffers-Daller, 2014; Bayram et al., 2017).

The heritage speakers and the L2 learners in our study are comparable in patterns of language dominance and in proficiency in the two languages, but differ in age and context of acquisition of the weaker language. We investigate whether these admittedly confounded variables (age and context acquisition of the weaker language) play a role in the morphological competence of bilinguals with similar dominance profiles. Given differences in timing, context and modality (auditory, written) of input experience, the question of whether and how heritage speakers and L2 learners differ in their linguistic knowledge continues to generate intense theoretical, empirical and practical interest (Santos and Flores, 2016; Perpiñán, 2017). Early acquisition and language experience gives heritage speakers a clear advantage compared to L2 learners when it comes to phonetics and phonology. Research has consistently shown that heritage speakers perform more native-like than L2 learners in phonological perception and production (Chang et al., 2011; Lukyanchenko and Gor, 2011; Chang, 2016). However, similar advantages for heritage speakers over L2 learners in morphosyntax have been less consistent, and the results in this area are more variable (Au et al., 2002; Foote, 2010). AoA is confounded with experience and context of learning in these two groups. Language experience, which includes amount and nature of input, is very relevant for the acquisition and mastery of morphology. Unsworth et al. (2014) attempted to disentangle the role of AoA from the role of input in Greek-English and Dutch-English bilingual children with different onsets of bilingualism and diverse language experiences and found a weak effect for AoA and a stronger effect of cumulative length of exposure with the acquisition of gender marking in nominals. Both L2 learners and heritage speakers are exposed to less input in the L2/heritage language than monolingually-raised children and balanced bilinguals who use the language more frequently and consistently. Yet heritage speakers may have more cumulative exposure to the weaker language than L2 learners because they were exposed to it earlier. If the acquisition of morphology is largely influenced by input factors, L2 learners and heritage speakers may show similar accuracy patterns on morphology. But if timing of input (i.e., AoA) determines the outcome of morphological acquisition instead, as it seems to impact phonology, heritage speakers may be more accurate with morphology than L2 learners. Because findings on morphology with respect to this question in adult heritage speakers and L2 learners have been inconclusive, more research on different languages and with different morphological patterns is warranted.

Our study contributes to this critical debate and is unique in examining morphology in Hindi, an understudied language. We focus on case morphology because case marking is very vulnerable to erosion in heritage languages in general (Benmamoun et al., 2013; Putnam and Sánchez, 2013; Kim et al., 2016), and is similarly difficult to acquire for L2 learners whose L1 does not mark case overtly (Papadopoulou et al., 2011; Baten and Verbeke, 2015; Baten et al., 2016). Hindi is a split-ergative language with a complex system of morphological case marking, and presents a challenging learning task for bilinguals whose dominant language has a nominative-accusative case pattern and does not mark morphological case overtly, like English. By probing into the syntactic and semantic distribution of the case particles -*ne* (ergative) and -*ko* (accusative, dative) in oral production and in an acceptability judgment task (AJT), we investigate how the complexity of morphological form-meaning mappings interacts with limited input factors and AoA in contributing to the acquisition of Hindi case morphology.

### Morphology in the Weaker Language

In both L2 acquisition and heritage language acquisition, morphology seems to be a bottleneck compared to other areas of the grammar (Slabakova, 2008; Montrul, 2018): it is difficult to acquire, easy to lose, and displays variability in L2 learners and heritage speakers. Following Distributed Morphology (Halle and Marantz, 1993), Lardiere (2009) explained how morphological knowledge may be acquired and computed during L2 learning. Language learners must assemble the lexicon of a language by associating lexical items (affixes, stems) with the specific formal features that the language selects from the inventory provided by Universal Grammar (Chomsky, 2001). Morphemes consist of features and feature bundles that encode phonological, semantic (+ interpretable) and syntactic (- interpretable) information, and languages assemble and combine features such as [+/- wh], [+/- plural], [+/- definite] in lexical and functional categories in different ways. Learners must learn which morpholexical forms and their allomorphic variants express which specific syntactic and semantic features, as well as the contextual conditions under which such morphological forms are realized overtly or as zero marking. Our study considers three factors that contribute to how fast and accurately bilinguals whose weaker language is an L1 or an L2 correctly reassemble and reconfigure features in the target language: (1) dominant language transfer, (2) the nature of the morphology itself (i.e., complexity assembling semantic and syntactic features of morphemes, and the transparency of form-meaning mappings), and (3) AoA of the weaker language.

It is well-established that in L2 acquisition, the dominant L1 guides and constrains the acquisition of the L2 (Schwartz and Sprouse, 1996). Morphological variability often occurs if the learner has not acquired the relevant abstract features or their values, or a given feature may be part of their L1 grammars but the learner lacks the relevant knowledge of the conditions for expressing the given feature in the L2. L2 learners look for morpholexical equivalents of their dominant language (the L1) features in the L2, assuming initially that L2 values are the same as in their L1 (Montrul, 2001). Development proceeds when learners are able to determine how to assemble the lexical items of the L2, by reconfiguring the feature values in lexical items and functional categories from their L1 to those of the L2 in cases where these are different (Lardiere, 2009).

For example, a type of case marking, Differential Object Marking (DOM), is frequently omitted in L2 and heritage language acquisition (Montrul, 2011). In Spanish, animate and specific direct objects are obligatorily marked with the preposition “a” (*Juan vio a María* “Juan saw María”). In Turkish, specific direct objects are marked with the accusative affix -*(y)I* (*Ayşe adam-ı gördü* “Ayşe saw the man”), and we will see that Hindi is similar to Turkish. Let’s assume that Spanish DOM bundles two features in the lexical item “*a*” [+ animate, + specific] whereas Turkish -*(y)I* bundles only one [+ specific]. Turkish-speaking learners of Spanish (Montrul and Gürel, 2015) have been shown to be more successful than English-speaking learners of Spanish (Guijarro Fuentes, 2012) at acquiring the feature specifications of DOM in Spanish. This is because Turkish-speaking learners had to only add the new [+ animate] feature of Spanish to their L2 Spanish representation, whereas the English-speaking learners had to build the representation for DOM in L2 Spanish anew, with its feature specification [+ animate, + specific]. Like Spanish, Romanian also bundles the features [+ animate, + specific] in the lexical item *pe* (the DOM marker in Romanian), and Romanian-speaking learners of Spanish have been shown to exhibit native-like acquisition of Spanish DOM (Montrul, in press). These studies provide clear evidence of L1 influence in the L2 acquisition of case morphology. As in L2 acquisition, transfer from the majority (stronger) language to the heritage (weaker) language is quite common in heritage speakers. The erosion and simplification of case found in Russian (Polinsky, 2006), in Spanish (Montrul and Bowles, 2009) and in Korean heritage speakers (Kim et al., 2016) in the United States could partially be due to the fact that English does not mark morphological case.

In addition to dominant language influence or transfer, the syntactic and semantic composition of morphemes and feature assembly is another likely cause of difficulty in L2 learners and heritage speakers. Morphological complexity has been linked to the number of elements making up individual morphemes and morphological systems (Pallotti, 2015) and the morphological computations that need to be performed under communicative pressure (Lardiere, 2016). Hawkins and Casillas’s (2008) Contextual Complexity Hypothesis predicts that the probability with which an inflectional morpheme will be omitted by early-stage L2 English learners is a function of the number of contextual dependencies to be calculated. For example, there are more steps in the computation of the English 3^rd^ person singular present subject-verb /-s/ than in the computation of -*in*g or -∅ (a bare verb), which may explain why learners are more likely to omit -*s* than to omit -*ing*. Complexity can also be related to the syntactic and discourse distribution of morphemes. Laleko and Polinsky (2016) found that L2 learners and heritage speakers of Korean and Japanese found morphological case markers that involve semantic and discourse computation (marked with topic) more difficult than case markers governed by syntactic constraints (nominative case).

Functional and cognitive approaches focus on the critical importance of processing for computing the mappings between concepts and morphemes from the input. Assuming an emergentist perspective, O’Grady (2008) and O’Grady et al. (2011) maintain that the language processing system has a key role in establishing what is initially acquired, what is subsequently retained or lost, and what is never acquired in the first place when considering how salience, frequency and transparency facilitate the establishment and strengthening of form-meaning mappings at the word and morpheme levels. Salience refers to acoustic prominence (-*ing* is more prominent and audible than -*s*); transparency to the one-to-one relationship between form and meaning, regularity to consistency and predictability in allomorphy involved in paradigms, and frequency to number of instances (types and tokens) in the input (see also Goldschneider and DeKeyser, 2001). The phenomena that are most susceptible to partial acquisition in heritage languages are those for which the form-meaning mappings are difficult to establish, either because the acoustic salience of a morpheme is weak (O’Grady et al., 2011), or because the precise semantic function or syntactic distribution may be difficult to figure out (Chung, 2016). These mappings require high frequency instantiations in the input, a condition that is not often met in heritage language and L2 learning. Longitudinal studies of child heritage speakers have found that when frequency and amount of input in the heritage language decrease with the shift to the majority language, inflectional morphology is very vulnerable at a young age (Silva-Corvalán, 2014), and instability persists into adulthood (Silva-Corvalán, 1991). In sum, both formal and functionalist approaches account for morphological errors and identify potential sources of difficulty related to the complexity of form-meaning mappings, entrenched knowledge of another language, and processing computations.

Another common explanation for persistent morphological variability in L2 acquisition concerns age effects. Johnson and Newport (1989) argued that the non-native acquisition of morphology in L2 learners was related to biologically-determined maturational effects on input processing mechanisms. Children are eventually better at mastering morphology because of their limited processing abilities compared to adults (the Less is More Hypothesis). Since then, a prevalent stance has been that L2 learners’ inability to (1) acquire features and abstract grammatical categories not instantiated in the L1 (Tsimpli and Dimitrakopoulou, 2007; Hawkins and Casillas, 2008), (2) achieve integrated knowledge of morphology (Jiang, 2004), and (3) process morphology like native speakers (Silva and Clahsen, 2008) is related to AoA after puberty (DeKeyser and Larson-Hall, 2005; Abrahamsson and Hyltenstam, 2009; Granena and Long, 2013). Others contend that ultimate attainment in L2 acquisition, and inability to reach native norms in all linguistic domains, is more readily explained by input and experience (Bialystok, 1997). Age effects are also relevant to explain the loss and weakening of their L1 in heritage speakers (Montrul, 2008): the younger the AoA of the majority language, the more likely the non-native acquisition of the heritage language (Yeni-Komshian et al., 2000), although input and experience also play a role (Jia and Aaronson, 2003). Heritage speakers contribute a different and unique angle on age effects because they illuminate how, despite L1 exposure since birth, restricted input in later childhood and adolescence greatly impacts the ultimate attainment of the heritage language in adulthood, especially at the morphological level.

## Case Marking in Hindi

Case marks thematic roles (agent, patient, goal) linked to syntactic positions (subject, object, indirect object), and there is cross-linguistic variation in case systems with respect to how different languages mark overt case. Some languages present a nominative-accusative pattern and others an ergative-absolutive pattern, as illustrated in Table 1.

**Table 1:** Case systems.

Nominative-accusative system		Ergative absolutive system	
Nominative case	Accusative case	Ergative case	Absolutive case
A	O	A	O
S			S

*A = Subject of transitive verb, S = Subject of intransitive verb, O = Object (of transitive verb).*

Nominative-accusative languages (e.g., Spanish, English, Russian, Greek, and German) generally mark subjects of transitive and intransitive verbs with nominative case, and objects with accusative case. Ergative-absolutive languages (e.g., Inuttitut, Dyirbal, and Basque) mark subjects of transitive verbs with ergative case. Subjects of intransitive predicates and objects of transitive predicates are marked with absolutive case (Butt, 2006). Very few studies have investigated the acquisition of languages with ergative-absolutive patterns (Bavin and Stoll, 2013) and the present study provides new empirical evidence from Hindi. As a split ergative language, Hindi behaves morphologically as an ergative language in certain contexts and as a nominative-accusative language in others (Dixon, 1994; Bittner and Hale, 1996). The morphological and syntactic status of the ergative case (as structural or inherent) in Hindi and in other languages compared to other cases continues to be a topic of lively theoretical debate in various frameworks (Marantz, 1991; Davison, 2004; Anand and Nevins, 2006; Butt, 2006; Woolford, 2006; Keine, 2007; Coon, 2013). Our study is strictly concerned with the morphological expression of these cases and less with their syntactic status (structural, non-structural), or the consequences of one particular syntactic analysis over another. Specifically, we focus on the acquisition of the syntactic and semantic conditions for the morphological expression of the particle-*ne*, which marks ergative subject, and the particle -*ko*, appearing with all indirect objects, some subjects, and some direct objects. Therefore, we adopt a morphological account.

The ergative split in Hindi is conditioned by perfectivity. Ergative marking can only appear on subjects of transitive-perfective verbs as in (1), which shows the ergative particle -*ne* on the subject *Nikhil*. In addition, the object can be absolutive (i.e., no overt case and controlling verbal agreement). In perfective clauses, ergative subjects tend to be interpreted as agentive, or to have volitional control (In all other cases, the subject is zero marked, i.e., nominative). Example (2) has a verb in the imperfective, and the subject carries nominative case because imperfective predicates cannot license ergative marking on the subject. Example (3) is ungrammatical because the verb is intransitive (and perfective), and intransitive verbs do not license ergative marking -*ne* on the subject.

**Table:** 

(1)	Nikhil-ne	akhbaar	paRh-ii	hai.
	Nikhil.MSg-Erg	newspaper.FSg.Nom	read-Perf.FSg	be.Pres.3Sg
	‘Nikhil has read the newspaper.’			
(2)	Nikhil	akhbaar	paRh-taa	hai.
	Nikhil.MSg.Nom	newspaper.FSg.Nom	read-Impf.MSg	be.Pres.3Sg
	‘Nikhil reads the newspaper.’			
(3)	^∗^Bela-ne	ghaNToN	dauR-ii/-aa.	
	Bela.FSg-Erg	for.hours	run-Perf.FSg/Perf.MSg	
	‘Bela ran for hours.’			

Case marking in Hindi interacts with verbal agreement. Main verbs and auxiliaries can agree with the subject (S-V agreement), with the object (O-V agreement), or with neither (default agreement). The verb agrees with nominative subjects. When subjects are ergative or dative the verb agrees with the nominative/absolutive object. If the subject and the object are overtly marked with ergative or accusative or dative, the verb shows default, masculine singular agreement. Object agreement marks number and gender, while subject agreement marks number, gender, and person. We do not discuss agreement in this study, but see Montrul et al. (2012).

The other particle we investigate, -*ko*, appears with some direct objects, with indirect objects and with dative subjects. With direct objects, Hindi shows differential object marking (or DOM) (Aissen, 2003; de Swart and de Hoop, 2007), which is triggered by animacy (+ human) and specificity. Human, definite and specific direct objects must be overtly marked with -*ko*, as in (4), and are ungrammatical without -*ko* marking (With non-human animates, -*ko* is optional depending on the animal).

**Table:** 

(4)	AaSaa-ne	Niiraj-ko	rokaa.
	Asha-Erg	Niraj-DOM	stopped
	‘Asha stopped Niraj.’		
			(cf. **^∗^**AaSaa-ne Niraj rokaa)

Human indefinite specific objects can be optionally *-ko* marked, as in (5).

**Table:** 

(5)	Sudhaa	ke	gharwaaloN-ne	us-ke liye	laRke
	Sudha	of	relatives.MPl-Erg	her-for	boys.MPl
	dekhe(dekhaa)				
	saw.MPl(saw.MSg)				
	‘Sudha’s relatives saw boys for her (for marriage).’				
					(laRkoN-ko)
					(boys.MPlObl-DOM)

With inanimate objects, -*ko* signals specificity. Inanimate, direct objects can be optionally marked with -*ko*, as in (6). If the object is -*ko*-marked it is interpreted as definite or specific; if it is unmarked it is non-specific.

**Table:** 

(6)	Aashaa-ne	rikshaa(/rikshe ko)	rokaa
	Asha-Erg	rikshaw (rikshaw DOM)	stopped
	‘Asha stopped a/the rickshaw.’		

In general, non-specific or indefinite inanimate objects are mostly unacceptable with -*ko* marking, as in (7), but the acceptability of -*ko* in these cases has to be evaluated in context.

**Table:** 

(7)	**^∗^**Sudhaa-ne	ek	caTTaan*-ko*	dekhaa.
	Sudha-Erg	a	rock-DOM	saw
	‘Sudha saw a (non-specific) rock’			

In sum, following Mohanan (1993), Aissen (2003), de Swart and de Hoop (2007), Dayal (2011), López (2012) and the judgments of the Hindi-speaking authors of this study, we assume these generalizations for -*ko* marking:

(1) -*ko* is obligatory with personal pronouns and human proper names (and optional with non-human animates).
(2) Definite, specific human DPs in object position take -*ko*.
(3) -*ko* is optional elsewhere (but depends on context)

-*ko* is the overt morphological expression of accusative case and carries the feature [+ specific] when it attaches to (inanimate) direct objects. Hindi DOM is primarily triggered by animacy (+ human), which is an inherent feature of nouns, and also by specificity (de Swart and de Hoop, 2007), which is determined contextually.

Additionally, dative -*ko* can mark indirect objects (goals, beneficiaries), as in (8), and dative subjects (experiencers), as in (9). The marking of indirect objects and dative experiencers with -*ko* is obligatory, irrespective of animacy, definiteness or specificity. Table 2 summarizes the distribution of the Hindi case particles discussed.

**Table 2:** Summary of the Hindi case particles examined in this study.

Particle	-*ne*	*-ko_1_*	*-ko_2_*	*-ko_3_*
*m-case*	Ergative	Accusative	Dative	Dative
Grammatical relation	Subject	Direct object	Indirect object	Subject
Thematic role	Agent	Patient/theme	Goal/beneficiary	Experiencer
Syntactic features	+ Subject + Oblique	- Subject + Oblique +α	- Subject + Oblique - α	+ Subject + Oblique
Semantic features	+ Perfective + volitionality	+ Human + Specific		- Perfective
Case valued in^1^	Perfective T	Transitive V	Ditransitive V	Lexical V

*There are other case markers, such as instrumental -*se*, genitive *-k* and locative *meN/par*. ^*1*^This is according to Keine (2007).*

**Table:** 

(8)	Manu-*ne*	Niiluu *ko*	ticket	dii.
	Manu-Erg	Nilu-Dat	ticket	gave
	‘Manu gave a ticket to Nilu.’			
				(cf. ^∗^Manu-ne Niiluu ticket dii)

**Table:** 

(9)	Manu-*ko*	vah	film	pasand hai
	Manu-Dat	that	movie	likes
	‘Manu likes that movie.’			
				(cf. ^∗^Manu vah film pasand hai)

Keine (2007) presents a morphological analysis of Hindi split ergativity and the particles -*ne* and -*ko* within Distributed Morphology (Halle and Marantz, 1993) to account for why these markers sometimes are realized as zero. In Distributed Morphology, there is separation of syntax and morphology such that morphology is added post-syntactically. Affixes carry abstract syntactic and semantic features that can be more or less specified, and compete for lexical insertion post-syntactically depending on how well they match the formal semantic and syntactic features of the stem.

In Keine’s analysis, which we assume for our study, ergative- *ne* is syntactically licensed in T if the predicate is perfective, as described in Table 2. Accusative -*ko* is licensed in V of transitive predicates; dative -*ko* of indirect objects in V of ditransitive predicates. Morphologically, both -*ne* and -*ko* alternate with the null marker, and Keine captures these patterns of overt/non-overt case alternations by means of morphological “impoverishment rules.” In general, the overt markers are chosen in Hindi, but in certain contexts features are deleted, only allowing for the null or zero marker to be attached. For example, the impoverishment rule for ergative in (10) states that if the subject of a transitive verb is in an imperfective clause, then the case realization is zero instead of -*ne*, as in example (2):

**Table:** 

(10)	[+ subject] → ø/[-perfective]		

The impoverishment rule for accusative -*ko* states that if a direct object is inanimate and non-specific, the morphological case is realized as zero instead of -*ko*, as in example (2).

**Table:** 

(11)	[+ oblique] → ø/[-human, -specific, + α]		

The contextual features of these impoverishment rules capture the principles underlying the alternations between overt and zero markers, therefore giving rise to split ergativity as a morphological phenomenon. Keine (2007) does not discuss the -*ko* of dative experiencer subjects, other than saying that it is a special case because this type of -*ko* is lexically determined and does not alternate with zero. We assume that dative subject -*ko* is licensed lexically by the experiencer feature of the subject and by the lexical V (Davison, 2004).

To summarize, the acquisition of ergativity raises questions about how monolingual and bilinguals identify the alignment patterns from the input and mark them correctly with ergative, nominative or absolutive case morphology. Not only does Hindi present both ergative-absolutive and nominative-accusative case patterns, but some cases can have multiple morphological realizations (overt/zero) and form-meaning mappings (one-to-one, as with -*ne*, and one-to-many, as with -*ko*). Case markers may not always be easy to perceive in the input: they vary in syntactic and semantic distribution and in frequency. Learners must implicitly perform distributional analyses of the input to figure out the structural differences between -*ko* as a marker of animacy with human direct objects and specificity with inanimate ones (DOM), but as an obligatory dative marker of *all* indirect objects and dative experiencers. This apparent variability in the Hindi system certainly presents a learning challenge for the acquisition and maintenance of case marking. As stated earlier, morphemes that are more frequent, map consistently to one meaning, and do not alternate with zero, are easier to acquire than morphemes that are less frequent in the input, map to more than one meaning, and alternate with zero (Kempe and MacWhinney, 1998; Goldschneider and DeKeyser, 2001; MacWhinney, 2008; O’Grady et al., 2011). On a formal account, morphemes that bundle more semantic and syntactic features and require multiple morphological computations are more likely to be omitted than morphemes requiring less steps in the computations (Hawkins and Casillas, 2008; Lardiere, 2016).

Paradoxically, languages that have rich morphology are easier to acquire than languages with sparser morphology, because the input provides many cues for morphological acquisition (Yang, 2002). But when input to the language is more restricted in terms of overall quantity and frequency, it will affect the abundance of morphological cues available and thus the degree of acquisition of morphology. Empirical evidence suggests that child learners of Hindi in India acquire and master ergative case marking relatively early while L2 learners and heritage speakers whose dominant language is English exhibit difficulty with accusative and ergative marking. Narasimhan (2005) studied three Hindi children in New Delhi, ages 1;7–3;9. The children made some omission errors with -*ne* but did not overgeneralize-*ne* to other predicates, and by the end of the observation period they showed between 80 and 100% accuracy on ergative marking. Under Keine’s (2007) analysis, the children have correctly learned the impoverishment rule of -*ne* deletion with imperfective predicates. Hindi is a null subject language and subjects are frequently dropped, as Narasimhan (2013) confirmed in the adult speech these children received, so the fact that Hindi children are so accurate at such an early age suggests that Hindi children are sensitive to perfective marking on the verb as a cue to ergativity. With respect to Hindi as a heritage language, Montrul et al. (2012) examined knowledge of case and agreement in oral narratives and grammaticality judgments. The Hindi heritage speakers produced and accepted ungrammatical sentences with omission of -*ne* and -*ko* with human specific direct objects, while the baseline Hindi-speaking adult immigrant group hardly omitted case markers in production or accepted ungrammatical sentences with omission in the judgment task. If overt -*ko* and ergative -*ne* marking is the default in these cases, following Keine’s analysis, perhaps influence of English, which does not have overt case marking, is what underlies the high incidence of zero marking in bilinguals whose Hindi is the weaker language. Two early studies on the L2 acquisition of Hindi by English-speaking learners (Hansen, 1986; Lakshmanan, 1999) reported comprehension errors with subject and object relative clauses because the learners ignored (did not process) case marking (accusative -*ko*). Baten and Verbeke (2015) and Baten et al. (2016) found that Dutch L2 learners of Hindi have difficulty with ergative and DOM marking in oral production. While there is some independent evidence that the acquisition of Hindi morphology is problematic for both heritage speakers and L2 learners of Hindi, no study has directly compared the nature of L2 learners and heritage speakers’ difficulty using the same methodology.

## The Study

Our study investigates knowledge of morphological ergativity in Hindi (accuracy on -*ne*) and -*ko* marking with different NPs (dative subjects, direct object, and indirect objects) in English–Hindi bilinguals with different dominance patterns and levels of proficiency in Hindi, guided by the following research questions and hypotheses:

(1) Does pattern of bilingual balance and proficiency in Hindi relate to knowledge of case marking in Hindi–English bilinguals?
(2) In bilinguals with Hindi as the weaker language, does their knowledge of case marking differ as a function of AoA of Hindi (early in heritage speakers, late in L2 learners)?

Balanced bilinguals with higher proficiency in Hindi are expected to have more native-like knowledge of case marking in Hindi than bilinguals for whom Hindi is the weaker language. As for differences between L2 learners and heritage speakers, assuming that case marking in Hindi is acquired by age 3 (Narasimhan, 2005, 2013), if heritage speakers of Hindi acquired case morphology early in a naturalistic setting and *retained it*, they may have an advantage in overall accuracy over L2 learners with acquisition of Hindi in adulthood, even if input and use of Hindi decreased as the bilinguals got older. But if limited input and use of Hindi beyond early childhood contributed to heritage speakers not fully learning or forgetting the syntactic and semantic features of morphemes, no advantages over L2 learners are expected.

Given the specific complexity of the Hindi case system with respect to English, the dominant language, two other questions we examine are as follows:

(3) If the unbalanced bilinguals make morphological errors in Hindi, will there be more omission or overgeneralization errors of the -*ne* and -*ko* particles?
(4) Since -*ko* has multiple functions in Hindi, is the morphological realizations of -*ko* marking with direct object, indirect objects and dative experiencers more difficult to master in some syntactic contexts than in others?

Lardiere’s (2009) Feature Reassembly Hypothesis is about linguistic representations whereas O’Grady et al.’s (2011) emergentist approach prioritizes the role of input and processing. These two models emphasize different aspects of the learning problem: what needs to be acquired (features and morpholexical forms) and on the basis of how it is acquired (noticing cues in the input). Both proposals make similar predictions regarding difficulties with different morphological markers, but for different reasons. We assume that ergative -*ne* and dative-experiencer -*ko* (*ko*_3_ in Table 2) are linked to agents of perfective predicates (ergative subjects) and experiencers of psychological predicates with stative verbs (dative subjects), respectively. Accusative -*ko* (*ko*_1_ in Table 2) is subject to NP constraints on definiteness and specificity with human and inanimate objects. Under certain semantic conditions ergative -*ne* and accusative -*ko* “appear” optional (realized as zero) when impoverishment rules apply (Keine, 2007). We further assume that -*ko* marking with indirect objects (-*ko_2_* in Table 2) encodes syntactic features but no additional semantic features, being less structurally complex than accusative *ko*_1_. Because it is consistently expressed as -*ko* (never zero) and most often refers to human goals/recipients, dative -*ko* with indirect objects is more reliable for learning than accusative -*ko*_1_.

For Keine’s (2007) analysis, *ne*-marking is the default for subjects and -*ko* marking is the default for human, specific direct objects: impoverishment rules in (10) and (11) apply with intransitive and imperfective predicates (leading to split ergativity) and when direct objects are inanimate and/or non-specific (DOM). If errors are observed, there will be overgeneralization (rather than omission) of -*ne* to intransitive imperfective predicates and of -*ko* to inanimate and non-specific objects, respectively. But since the participants are bilingual and their stronger language is English, which does not have ergative case and DOM, dominant language transfer in this case may lead to significant more omission of the markers rather than to overgeneralization.

With respect to the specific complexity of the markers, we hypothesize that if L2 learners and heritage speakers make errors, these will be determined by the syntactic and semantic complexity of the markers. According to the Feature Reassembly Hypothesis, markers that bundle more semantic features will be more difficult to master than markers that bundle fewer features or only one. We thus expect higher accuracy with the -*ko* of indirect objects (*ko_2_*) than with ergative -*ne*, the -*ko* of dative experiencers (*ko_3_*) and the -*ko* of specific direct objects (*ko_1_*). From an input-based perspective, -*ko_2_* with indirect objects is a more reliable cue than -*ko_1_* with direct objects because all indirect objects are marked with -*ko* (i.e., it is not subject to any impoverished rule) whereas *some* direct objects are marked with zero. Therefore, this theoretical position also predicts higher accuracy on -*ko* marking of indirect objects than on the other three markers. Dominant language transfer can also account for omission of -*ko* with dative subjects, since English has nominative subjects with psych verbs. -*ko* marking with indirect objects is again predicted to be the easiest to be acquired because it is marked by a preposition (*to*) in NP PP configurations in English.

### Participants

Bilingual dominance in this study was determined by the linguistic and biographical characteristics of the bilinguals recruited, including AoA of the languages, place of upbringing, place of current residence, as well as specific linguistic measures of proficiency in Hindi. A total of 73 young adult Hindi–English bilinguals participated in this study. Based on language learning experience, place of current residence (United States vs. India) and self ratings on Hindi and English, the participants were grouped in three groups: a balanced bilingual group tested in India (the baseline group) (*n* = 23) and two groups of English–Hindi bilinguals dominant in English (26 Hindi heritage speakers, 24 L2 learners of Hindi). All participants completed an extensive language background questionnaire, a written interview protocol that elicits short answer questions about demographic and biographical information, including information about the languages spoken at home, the activities performed in each language, the participants’ current and past exposure to and use of Hindi and English at home and in other contexts (including school, travel), regular presence of grandparents, presence of older siblings, the languages used by parents with the heritage speakers at different times in childhood and the languages used by the heritage speakers with the parents. The questionnaire contains questions with Likert-scales eliciting information about perceived abilities in the two languages by skill (speaking, listening, reading, and writing), and estimates of quantity of input from estimates of the percentage of each language addressed to the participant, and the estimated amount of Hindi used by the participant beyond the home (TV, reading, internet, extracurricular activities, church), and with different interlocutors (parents, siblings, grandparents, friends, other) who speak Hindi. Many of the questions in this questionnaire were not relevant for the L2 learners of Hindi, who were all born and raised in English-speaking homes in the United States.

The heritage speakers of Hindi, mean age 21.5 (range: 18–25), were recruited in Illinois and in New Jersey. They were simultaneous bilinguals exposed to English and Hindi in early childhood, born in the United States to highly educated Hindi-speaking parents (both father and mother). Due to the multilingual situation in India, the parents spoke both English and Hindi in addition to a regional South-Asian language (Punjabi, Gujurati, Marathi, Telugu, Tamil, among others). Some of these languages are ergative and others are not, but as we will see in the results, knowledge of these languages did not lead to variability with ergative marking in this group. Most of the heritage speakers (*n* = 20) spoke English and Hindi before age 5 and the rest (*n* = 6) spoke only Hindi. All Hindi heritage speakers were schooled in English and 18 indicated that they received from 2 to 10 h of instruction per week in Hindi as a heritage or foreign language in elementary and middle school through their parents. Use of Hindi during their lifetime was mostly with the parents and to a more limited extent with siblings. At present, 13 preferred to use English exclusively, while the rest would use more English than Hindi, depending on the situation. The heritage speakers were not taking Hindi classes at the time of testing, but they had all traveled to India at least once. When asked how they felt about Hindi, 4 (18%) indicated it was their native language and 22 (82%) their second language. Their mean self-assessments indicates that the majority of individuals in this group perceived Hindi as their less dominant language, and their impressions is corroborated by the biographical and language use information collected with the questionnaire.

The L2 learners of Hindi were graduate and undergraduate students ages 21 to 37 (mean: 26.24) taking Hindi as a foreign language in Illinois. Their mean length of exposure to Hindi was 4.2 years (*range* 1 to 7). They were all native speakers of English and started learning Hindi between the ages of 18 and 29 (*mean* age: 22). The learners were enrolled in advanced classes three or four times a week, which focused on reading, writing and speaking skills through culture. Eleven had traveled to India for 2 weeks to 5 months. Reasons for studying Hindi ranged from professional and academic (46%) to personal fulfillment (54%).

A main issue when doing experimental studies with heritage speakers is the baseline (Montrul, 2016b), and this depends on the objective of the study. Because our goal was not to examine the intergenerational language transmission in immigrants (for a study of intergenerational transmission see Montrul et al., 2015), we did not use a group of first generation adult immigrants in this study, and we chose to compare instead the heritage speakers and the L2 learners to age and SES matched peers in India, who are also bilingual in English and Hindi. The Hindi speakers from India were young university-educated adults between the ages of 18 and 25 residing in Delhi. They were fluent bilinguals in Hindi and English and, like the parents of the heritage speakers, most of them also spoke another South Asian language (Punjabi, Gujarati, Marathi, Tamil and Telugu, among others). It was not possible to control for what type of other South Asian languages the speakers knew. Sixteen (70%) reported that Hindi was their native language and 7 (30%) their second language. As far as patterns of language use at time of testing, 7 (30%) used Hindi the most in every day life, 4 (17%) used more English than Hindi, and the rest (52%) used the two languages on a daily basis.

The background questionnaire included self-rating scales on Hindi and English. Participants rated on a scale from 1 (none) to 5 (native ability) their overall perceived ability in English and in Hindi, in receptive (listening, reading) and productive (speaking, writing) skills. They also completed a written proficiency test, consisting of a cloze passage in Hindi with 40 blanks every seven words and three multiple-choice responses per blank (same cloze test was used in Montrul et al., 2012, 2015). This cloze task was created by one of the authors of this study and was piloted with native and non-native speakers of Hindi. Reliability statistics (Cronbach alpha) run on the responses of the cloze test yielded a coefficient above 0.80. Although we did collect information about the frequency of use of Hindi and English for all the speakers tested, following Montrul (2016a) we assessed dominance quantitatively, by combining the scores from the self-ratings and accuracy in the Hindi written proficiency measure. Reported amount of input and use of the language, an important dimension of dominance (Montrul, 2016a), largely corroborated the self-ratings and general Hindi proficiency scores.

Table 3 presents the self-ratings in each language for the three groups. Figure 1 shows the dominance patterns of the three groups based on the overall self-ratings. Comparison of mean self-ratings in English and in Hindi showed that the Hindi speakers in India self-evaluated their overall Hindi proficiency as high as their English [paired samples *t*-test: *t*(22) = 1.19, *p* > 0.05], and evaluated similarly their four skills in each language (all *p*s > 0.05). Therefore, they are considered balanced in English and Hindi bilinguals for this study. The heritage speakers and the L2 learners self-rated their English at native level and their Hindi significantly lower [paired samples *t*-tests: L2 learners *t*(23) = 13.515, *p* < 0.0001, heritage speakers *t*(25) = 8.17, *p* < 0.0001]. Thus, they considered unbalanced bilinguals for this study, with English as dominant language and Hindi as their weaker language. The L2 learners and the heritage speakers assigned lower ratings to their Hindi than the speakers in India (balanced bilinguals) (one way ANOVAs and Tukey *post hoc* tests, all *p*s < 0.0001). Except for speaking, which the L2 learners and the heritage speakers rated similarly (2.88 and 2.58, *p* > 0.05), the two groups differed on their assessments of reading, listening and writing skills (all *p*s < 0.05). The heritage speakers rated their listening skills higher (3.46) than the L2 learners (2.48), whereas the L2 learners rated their reading and writing skills (3 and 3.04) higher than the heritage speakers (1.88 and 1.65). The heritage speakers attended English only schools but many said they received instruction in Hindi during the elementary and middle school period through their parents, whereas the L2 learners learned to read and write the Hindi script in the classroom. This difference among skills within and between the two groups confirms the common profile of L2 learners and many heritage speakers in their weaker language.

**Table 3:** Mean self-ratings in Hindi and English language skills (1 = none-limited ability, 5 = native ability), *SD*s are in parentheses.

Language	Skill	Groups					
		Hindi speakers in India (*n* = 23)		Hindi heritage speakers (*n* = 26)		L2 learners of Hindi (*n* = 24)	
		Mean	*SD*	Mean	*SD*	Mean	*SD*
English	Reading	4.26	(1.09)	5	(0)	5	(0)
	Speaking	4	(1.04)	5	(0)	5	(0)
	Listening	4.30	(1.06)	5	(0)	5	(0)
	Writing	4.04	(1.06)	4.92	(0.27)	5	(0)
	Overall	4.34	(1.07)	5	(0)	5	(0)
Hindi	Reading	4	(1.12)	1.88	(0.95)	3	(0.83)
	Speaking	4.13	(0.96)	2.88	(1.21)	2.58	(0.97)
	Listening	4.43	(0.78)	3.46	(1.17)	2.58	(0.82)
	Writing	3.69	(1.25)	1.65	(0.79)	3.04	(0.80)
	Overall	4.65	(0.57)	3.26	(1.07)	3.12	(0.67)

**FIGURE 2:**
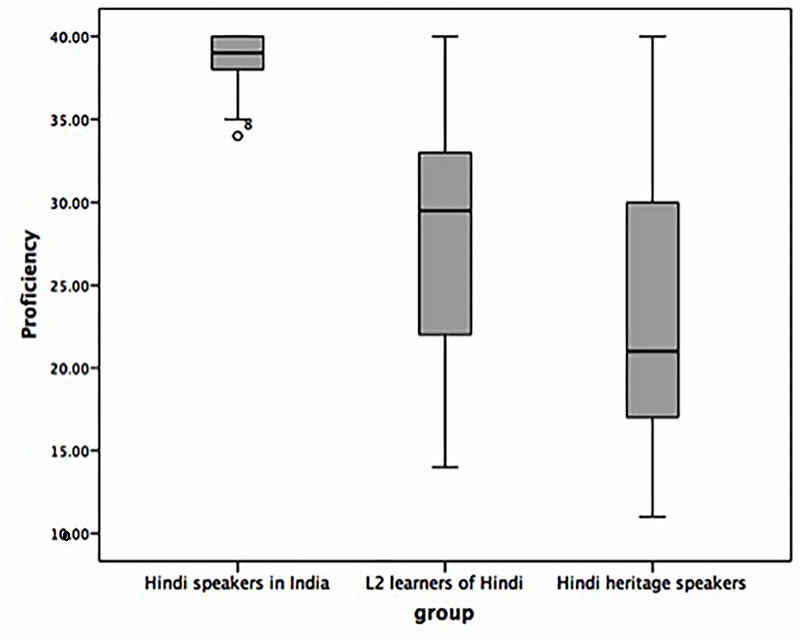
Mean distribution of scores in the written Hindi proficiency cloze test.

The results of the Hindi proficiency test in Figure 2 reflected similar differences between the three groups [one way ANOVA, *F*(2,72) = 32.08, *p* < 0.0001]. From a total maximum of 40 points, the mean score for the speakers in India was 38.56 (34–40, *SD:* 1.82), 24.11 (11–40, *SD:* 8.60) for the heritage speakers, and 27.91 (14–40, *SD:* 6.62) for the L2 learners. Multiple comparisons showed no statistical difference between the proficiency scores of the L2 learners and the heritage speakers (*p* > 0.05). The overall Hindi proficiency ratings and the scores on the written proficiency test correlated positively, *r* (two-tailed) for the L2 learners = 0.59, *p* = 0.001 and for the heritage speakers = 0.55, *p* = 0.006.

### Tasks

An oral production task and a bimodal AJT were used to assess knowledge of morphological case. The oral production task elicited differential object marking and dative case, and consisted of pictures with two participants and transitive verbs requiring animate (human) or inanimate objects and verbs that take dative subjects. The task included 35 sentences: 7 with dative subjects, 14 with human objects, 14 with inanimate objects. Participants were asked to describe the pictures using the past tense, with many opportunities to use perfective predicates. Therefore, we examined the production of ergative marking in the same task. Responses were audio-recorded, transcribed, and analyzed for correct suppliance, omission, or overgeneralization of the case markers -*ko* and -*ne*.

The AJT (Montrul et al., 2012) complemented the results obtained with elicited production. The AJT was bimodal, with stimulus presentation in visual and auditory modality. Since L2 learners tend to do better in written than in auditory tasks whereas heritage speakers do better in auditory than in written tasks (Montrul et al., 2008), the bimodal presentation was done to not advantage or disadvantage the unbalanced bilingual groups with respect to each other. There were 216 sentences (half target, half fillers, half grammatical, half ungrammatical/infelicitous) divided into 24 types, with 6–12 token sentences per type, depending on the structure. Sentence types included minimal pairs with correct and incorrect uses of ergative case and verb transitivity in simple and compound verbs, and sentence types with human animate and inanimate, specific/non-specific direct objects, indirect objects and dative subjects, where the presence and omission of the case marker -*ko* were manipulated, like the examples presented in (1) to (9). For the sentences testing ergativity, we only manipulated transitivity; we did not manipulate perfectivity due to the length of the test. However, perfectivity errors were evaluated in the oral task. Even though the stimuli consisted of minimal pairs (the same verbs and sentence structure with and without the relevant morphology), the sentences were presented in randomized order (not in pairs), and each sentence was judged independently. The AJT was administered through the web interface Survey Gizmo. Each sentence was presented in Hindi script and with an audio player below. Participants were instructed to read each sentence and play the sound file before rating each sentence on a 1–4 scale (1 = completely unacceptable to 4 = perfectly acceptable). The task was self-paced and did not measure reaction times (completion time was about 20–30 min). Participants could not go back and compare sentences: once a sentence was rated, it disappeared from the screen.

## Results

### The Elicited Production Task

Although the initial pool of L2 learners was 24, we have results for 19 learners. Two audio files were corrupted, and three learners who were not confident in their oral skills refused to complete the task. Transcriptions were coded for ergative *-ne* and accusative/dative *-ko* marking in obligatory contexts and for potential omission errors, and in non-obligatory contexts for potential overgeneralization errors. For ergativity, we coded presence or absence of -*ne* and its accuracy (correct/incorrect) based on transitivity (transitive/intransitive) and perfectivity (perfective-non-perfective) of all verbs. For-*ko* we coded presence or absence of -*ko*, its accuracy (correct/incorrect) based on type of NP (direct object, indirect object, dative subjects) and animacy of the direct objects (human animate, non-human animate, and inanimate). Transcriptions, coding and inter-rater reliability checks were done by two of the Hindi-speakers in our team. Since this was production, the number of relevant tokens produced would be different for each speaker.

Most of the participants responded in the past and used many instances of ergative case, but some did not use past or ergative marking. Therefore, the results are based on the number of participants in each group who produced ergative -*ne* marking in required transitive perfective contexts. The number of observations included in the analysis was 2210. There were very few instances of intransitive predicates with no ergative marking produced in the entire data, all correct (1 instance from 1 heritage speaker, 5 instances from 5 speakers from India and 3 instances by 3 L2 learners). We counted overgeneralization errors of -*ne* with sentences in non-perfective contexts (present, progressive, or imperfective). Several heritage speakers and L2 learners made omission errors with perfective predicates, as in (12) and (13), or overgeneralizations of -*ne* to imperfectives, as shown in (14) and (15).

**Table:** 

(12)	**^∗^sarah-φ**	eva	ko	khiiNc-aa	thaa
	Sarah	Eva	ACC	pull-Perf.MSg	Pst.MSg
	‘Sarah had pulled Eva.’				
					(Hindi heritage speaker)

**Table:** 

(13)	**^∗^bill-φ**	sara	ko	rulaa-yaa
	Bill	Sara	ACC	make.cry-Perf.MSg
	‘Bill made Sara cry.’			
				(L2 learners of Hindi)

**Table:** 

(14)	tom	**^∗^ne**	stephanie	ko	cup	kar	rahaa	hai
	Tom	Erg	Stephanie	ACC	pacify	do	Prog.MSg	Pres.Sg
	‘Tom is pacifying Stephanie.’						
								(Hindi heritage speaker)

**Table:** 

(15)	john	**^∗^ne**	esha	ko	uThaa	rahaa	hai
	John	Erg	Esha	ACC	pick	Prog.MSg	Pres.Sg
	‘John is picking Esha up.’						
							(L2 learner of Hindi)

The raw data were analyzed using binomial linear mixed-effects models (Jaeger, 2008) in R Core Team (2014) on categorical data (correct, incorrect), better suited to analyze categorical data and unbalanced data (Jaeger, 2008, p. 436). All independent variables were added to the model following a stepwise procedure, and subsequently, models containing interactions between factors were also incorporated to the analysis. In identifying the best-fitted model for our data, all nested models were compared using the function ANOVA. The most reliable model was chosen based on lowest AIC values.

The best model for ergativity marking included group (heritage speakers, L2 learners, speakers from India) and aspect (perfective, non-perfective) as fixed effects, and participants and items (random intercepts only) as intercepts. The dependent variable was accuracy on -*ne* marking. Table 4 shows accuracy on ergative -*ne* marking with transitive, perfective predicates and rates of omission, and overgeneralization of -*ne* to non-perfective predicates in oral production.

**Table 4:** Elicited Production Task.

Groups	*N*	Accuracy on -*ne* with transitive predicates	Total error rate	Omission of -*ne* with perfective predicates	Extension of -*ne* to imperfective predicates
Hindi speakers in India	23	99.1	0.9	0.02	0.00
Hindi heritage speakers	26	81.5	18.5	42.31	0.75
L2 learners of Hindi	19	76.5	23.5	19.41	62.5

*Percentage Accuracy on ergative -*ne* marking.*

The percentage accuracy of -*ne* and the total error rate was significant by group (β = 3.25, *SE* = 0.89, *z* = 3.639, *p* < 0.0001). There was a main effect for aspect (β = 3.52, *SE* = 0.37, *z* = -9.50, *p* < 0.0001) and an aspect by group interaction (β = -7.79, *SE* = 0.96, *z* = -8.079, *p* < 0.0001). Tukey *post hoc* comparisons revealed that overall accuracy on *ne*-was significant different (*p* < 0.05) between the three groups, but the interaction indicated that the heritage speakers omitted -*ne* (42.3%) with transitive perfective predicates more than the L2 learners (19.4%) (*p* < 0.0001). The error types were examined by omissions and overgeneralizations. Although instances of transitive imperfective predicates with -*ne* were very few in the data (total 18), the L2 learners produced significantly more overgeneralization errors with -*ne* (62.5%) with imperfective predicates than the heritage speakers (0.75%) (*p* < 0.0001). The heritage speakers showed the opposite pattern: when errors were made, these were more of omission than of overgeneralization.

Next, we analyzed the use of -*ko* marking with direct objects, which is obligatory if the object is human animate and specific. The number of observations included in the analysis was 1702. Most of the examples included names or referred to people (grandmother, hunter). Examples (16) and (17) are errors of omission of -*ko* with human objects.

**Table:** 

(16)	**^∗^Teacher-φ**	khuS	kar	rahe	haiN	chaatra
	teacher	please	do	Prog.MPl	Pres.MPl	students
	OBJ	V				SUBJ
	‘The students are making the teacher happy.’					
					(Hindi heritage speaker)	

**Table:** 

(17)	grandmother	**^∗^Albert**	laa-ii
	grandmother	Albert	bring-Perf. FSg
	SUBJ	OBJ	V
	‘The grandmother brought Albert.’		
			(L2 learners of Hindi)

We included sentences with inanimate objects but because Hindi does not have articles, the slides only listed the name of the object. Specific inanimate objects are marked with -*ko*; non-specific objects are unmarked in Hindi. So, if the participants chose to make the object specific, they would use -*ko* and if they made the object non-specific, they would not mark it with -*ko*. Sentences were not presented in context, so the use of -*ko* with inanimate objects was optional, depending on whether the participant meant the object to be specific or not, as in (18) non-specific unmarked and (19) specific, marked.

**Table:** 

(18)	Pati	patnii	**form**	bhar-eNge
	husband	wife	form	fill-Fut.MPl
	‘The husband and wife will fill a form.’			
				(Hindi heritage speaker)

**Table:** 

(19)	aadmii	ne	**chaate**	**ko**	uThaa-yaa
	man	Erg	umbrella	ACC	pick-Perf.MSg
	‘The man picked up the umbrella.’				
						(L2 learner of Hindi)

Finally, -*ko* marking is also obligatory with dative subjects, but L2 learners and heritage speakers produced omission errors with these predicates, as in (20) and (21).

**Table:** 

(20)	maaN	kii	madad	se	**^∗^ye-φ**	garv	huaa
	mother	of	help	with	he	proud	happen.Perf.MSg
	‘He became proud with mother’s help.’						
							(L2 learner of Hindi)

**Table:** 

(21)	**^∗^maaN-φ**	beTe	par	garv	ho	rahii	hai
	mother	son	at	proud	be	Prog.FSg	Pres.Sg
	‘The mother is feeling proud of her son.’						
							(Hindi heritage speaker)

Table 5 shows the percentage production of -*ko* marking by NP type (direct objects, indirect objects and dative subjects) and Table 6 shows production of -*ko* by the animacy of the direct object (Human, non-human animate, and inanimate).

**Table 5:** Elicited Production Task.

Groups	*N*	Direct object	Indirect object	Dative subject
Hindi speakers in India	23	100	100	98.96
Hindi heritage speakers	26	83.87	91.70	66.19
L2 learners of Hindi	19	88.23	93.55	86.04

*Mean percentage accuracy accusative/dative -*ko* marking by NP type.*

**Table 6:** Elicited Production Task.

Groups	*N*	Human	Non-human animate	Inanimate
Hindi speakers in India	23	100	100	100
Hindi heritage speakers	26	85.00	79.16	98.36
L2 learners of Hindi	19	86.38	76.92	100

*Mean percentage accuracy accusative-*ko* marking by animacy.*

We conducted two binomial mixed effects models with *ko*-accuracy as dependent variable. The first one included group and NP type as fixed effects, with participants and items as random effects. This model found a main effect for group (β = -1.91, *SE* = 0.68, *z* = 2.79, *p* < 0.01) and an NP type by group interaction (β = 2.20, *SE* = 0.49, *z* = 4.419, *p* < 0.0001). The main effect by group found that the L2 Hindi speakers were statistically significant from the L2 learners and the heritage speakers (*p* < 0.0001). The groups by NP type interaction found that the heritage speakers omitted -*ko* with dative subjects more than with direct and indirect objects (*p* < 0001). The second binomial fixed effects model included type of direct object and group as fixed effects with subject and items as random intercepts. Accuracy production of -*ko* was the dependent variable. This model found a significant main effect for animacy (β = 3.75, *SE* = 0.48, *z* = 7.67, *p* < 0.0001), no main effects for groups, and no interactions. Although the speakers from India performed at ceiling, there were no differences between the L2 learners and the heritage speakers in their overall accuracy: both groups omitted -*ko* with animate direct objects to the same extent (human: 16.13% heritage speakers, 11.77% L2 learners; and non-human: 20.84% heritage speakers, 23.08% L2 learners). There were very few overgeneralizations of -*ko* to inanimate objects. In general, the data in Tables 5, 6 show that heritage speakers and L2 speakers, whose weaker language is Hindi, omitted obligatory accusative case marking with human direct objects and dative case with dative subjects, unlike the speakers from India (balanced bilinguals).

Summarizing, with respect to ergative marking, there were more omission than overgeneralization errors of -*ne* for the heritage speakers. With accusative -*ko*, the two unbalanced bilingual groups produced omission errors with human animate objects to the same extent (-*ko* with non-human objects is more variable). The heritage speakers produced a few -*ko* marking errors with inanimate objects compared to the L2 learners and the speakers from India, but these cannot necessarily be considered errors if the descriptions were meant to be specific, since the sentences were not ungrammatical. Finally, the L2 learners and the heritage speakers omitted -*ko* with dative subjects more than with indirect objects.

### The Acceptability Judgment Task (AJT)

In the AJT, grammatical and ungrammatical sentences manipulating the markers -*ne* and -*ko* were judged on a scale from 1 to 4. Acceptability ratings were submitted to ordinal regression mixed effects models in R. One model included acceptability ratings as dependent variable, sentence type, grammaticality and group as fixed factors, and participants and items as random intercepts. The model found a main effect for group (β = 4.36, *SE* = 1.78, *t* = 2.45, *p* < 0.01), for sentences (β = -1.71, *SE* = 4.40, *t* = -3.887, *p* < 0.0001) and a group by sentences interaction (β = 7.15, *SE* = 1.98, *t* = 3.605, *p* < 0.0001). To investigate the group by sentences interaction further, we ran models on -*ne* and -*ko* sentences separately.

**FIGURE 3:**
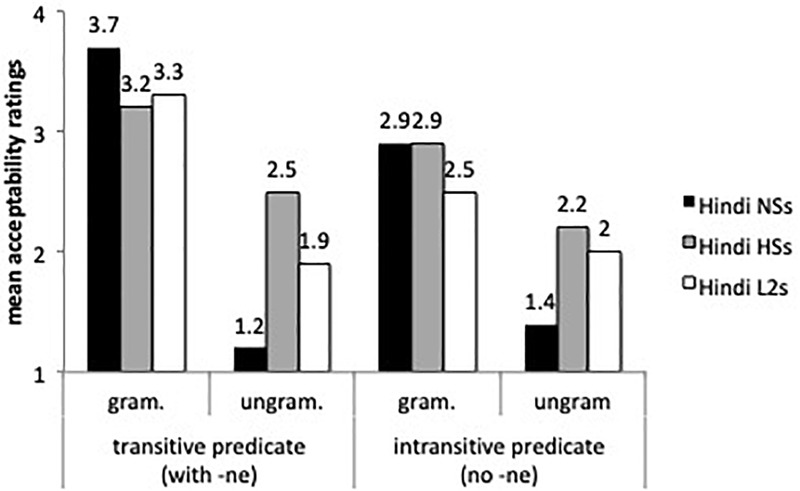
Mean acceptability ratings for ergative marking (-*ne*) with transitive and intransitive perfective predicates.

With respect to -*ne* marking (ergativity), we tested transitive and intransitive verbs, in both simple and compound verbs in the perfective form. Figure 3 displays the mean acceptability ratings for transitive and intransitive predicates.

A mixed effects model with group by transitivity and grammaticality showed main effects for grammaticality (β = -1.083, *SE* = 7.16, *t* = -15.116, *p* < 0.00001), and group (β = 4.81, *SE* = 1.05, *t* = -0.81, *p* < 0.0001), and a group by grammaticality interaction (β = 3.354, *SE* = 5.66, *t* = 5.924, *p* < 0.0001). The heritage speakers and L2 learners’ ratings were statistically different from those of the speakers in India (*p* < 0.001), but Tukey *post hoc* tests revealed no differences between the L2 learners and the heritage speakers (*p*s > 0.05). The group by grammaticality interaction indicated that the Hindi speakers from India, the heritage speakers and the L2 speakers differed in their ratings of ungrammatical sentences. The heritage speakers assigned higher acceptability ratings to ungrammatical sentences with transitive and intransitive predicates than the native speakers from India (β = -1.12, *SE* = 5.95, *t* = -18.932, *p* < 0.0001) and the L2 learners (β = 3.354, *SE* = 5.66, *t* = 5.924, *p* < 0.0001). As for omission of -*ne* with transitive perfective predicates, the results confirm the findings of the production task: the L2 learners and the heritage speakers assigned higher acceptability ratings to ungrammatical sentences with omission of *-ne* than the speakers from India, and the difference between the experimental groups’ ratings was not significant (all *p*s > 0.05). Heritage speakers and L2 learners were also more accepting of intransitive perfective predicates with -*ne* (i.e., overgeneralization errors) than the speakers from India (*p* = 0.01), according to Tukey *post hoc* tests, suggesting unstable knowledge of -*ne* marking in intransitive predicates as well. For the heritage speakers and for the L2 learners we conducted pairwise comparisons of the two ungrammatical sentences: transitive predicates without -*ne* (omission) and intransitive predicates with -*ne* (overgeneralization) and there were no statistical differences between the ratings for either group.

**FIGURE 4:**
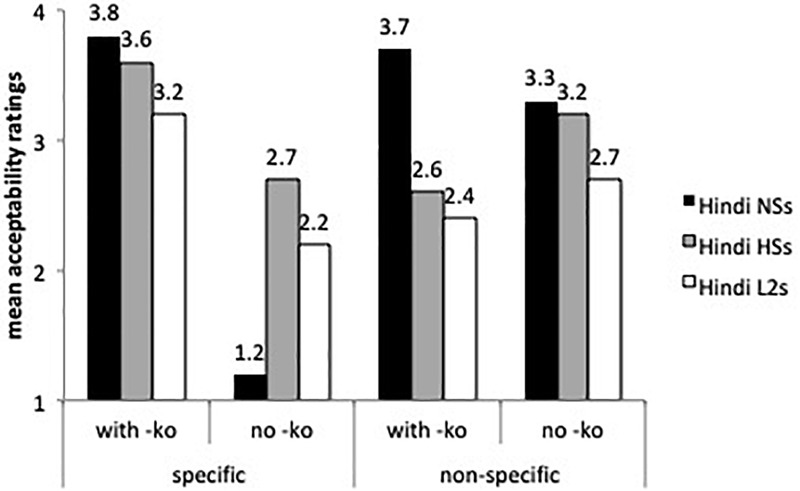
Mean acceptability on -*ko* marking with animate, specific direct objects.

Figure 4 depicts the results of -*ko* marking with animate (human) specific direct objects, which is required in all these cases.

According to the best-fitted fixed effects model ran on animate objects (with group, sentences and grammaticality as fixed factors and subject and items as random intercepts), the speakers from India differed significantly from the heritage speakers and the L2 learners (β = 0.98, *SE* = 0.19, *t* = 5.15, *p* < 0.0001). Human specific objects with -*ko* received overall higher ratings than human non-specific objects with -*ko* (β = 0.861, *SE* = 0.22, *t* = 3.849, *p* < 0.0001). There was a sentence type by group interaction: the L2 learners assigned lower acceptability ratings to grammatical sentences with -*ko*-marked human non-specific direct objects than the Hindi speakers from India (β = 0.41, *SE* = 0.20, *t* = -2.057, *p* < 0.01). Even though the three groups rated grammatical and ungrammatical -*ko* with human specific objects differently (the comparison of grammatical and ungrammatical sentences was significant at the *p* < 0.0001), the L2 learners and the heritage speakers were more accepting of -*ko* omission with human specific direct objects than the speakers from India (β = -1.356, *SE* = 0.35, *t* = -3.867, *p* < 0.0001), a result that confirms the omission errors found in the oral task. Other contrasts were not significant.

Figure 4 displays ratings on inanimate objects, both specific and non-specific, with and without -*ko* marking. The mixed effects model found a main effect for group (β = 0.73, *SE* = 0.16, *t* = 4.346, *p* < 0.0001), for sentences (β = 0.5069, *SE* = 0.22, *t* = 2.216, *p* < 0.01), and a sentence by group interaction (β = 0.44, *SE* = 0.17, *t* = 2.49, *p* < 0.01). As the speakers from India’s ratings show, -*ko* is more likely to be dropped with inanimate objects than with human specific direct objects (see Figure 3). The heritage speakers did not differ from the speakers from India with specific and non-specific inanimate objects, and the L2 learners were less accepting of -*ko* marking than the heritage speakers (β = -0.56, *SE* = 0.22, *t* = 2.48, *p* < 0.01) with specific inanimate objects. Although the speakers from India assigned higher ratings to inanimate non-specific objects with -*ko* marking than expected, our task presented sentences in isolation, and without context these sentences can be assumed to involve specific objects and hence acceptable.

**FIGURE 5:**
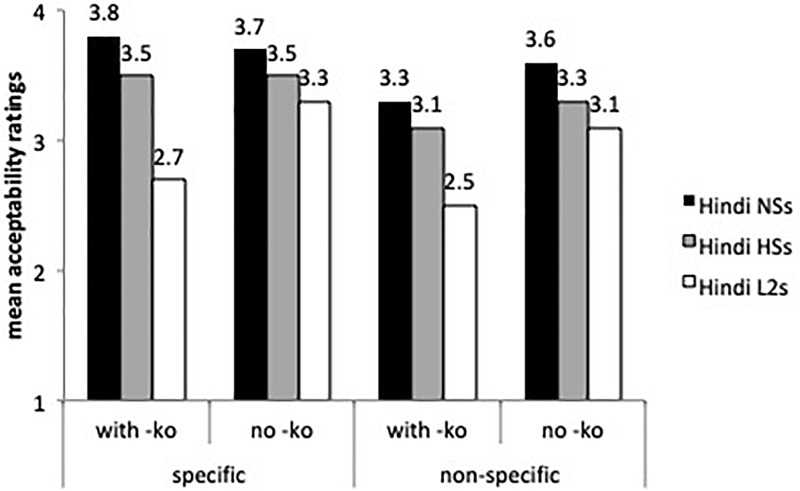
Mean acceptability on -*ko* marking with inanimate objects.

Recall that human, specific direct objects with no -*ko* are ungrammatical (omission error) (Figure 4) and in principle inanimate non-specific objects with -*ko* are ungrammatical (potential overgeneralization error) (Figure 5). The acceptability ratings on these sentences were not significant.

**FIGURE 6:**
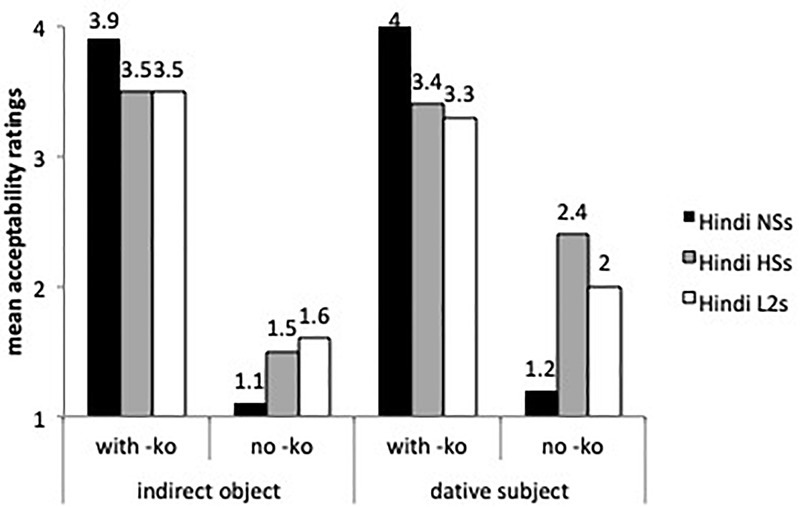
Mean acceptability ratings on indirect objects and dative subjects with and without -*ko*.

Figure 6 shows the acceptability of -*ko* marking with indirect objects and dative subjects. The mixed effects model performed on these sentence types found a main effect for group (β = 0.52, *SE* = 0.15, *t* = 3.316, *p* < 0.0001), by sentence type (β = -1.77, *SE* = 0.17, *t* = -10.125, *p* < 0.0001) and a group by sentence type interaction. All three groups seem to know that -*ko* is grammatical with these two sentence types. The heritage speakers and the L2 learners were more accepting of indirect objects (β = -1.108, *SE* = 0.19, *t* = -5.778, *p* < 0.0001) and of dative subjects without -*ko* (i.e., omission) (β = -0.95, *SE* = 0.18, = -5.158, *p* < 0.0001) than the speakers from India. The L2 learners and the heritage speakers did not differ from each other (Tukey tests non-significant).

**FIGURE 7:**
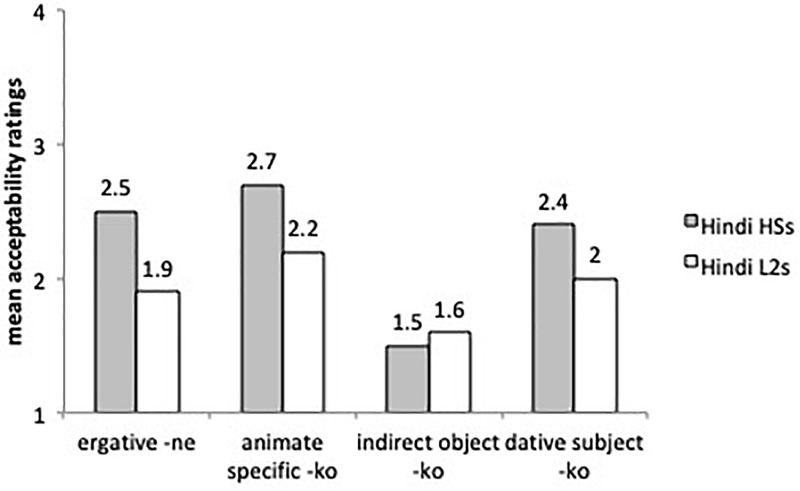
Mean acceptability ratings on ungrammatical sentences with omission of obligatory case marking.

Finally, Figure 7 compares the mean acceptability ratings of ungrammatical sentences with case omission (errors) with ergative subjects (-*ne*), animate specific direct objects (*ko*_1_), indirect objects (*ko*_2_) and dative subjects (*ko*_3_) by the two experimental groups. Per our hypothesis we expected lower ratings (i.e., less acceptance of omission) with indirect objects (*ko_2_*) than with ergative -*ne*, dative experiencers (*ko_3_*) and specific direct objects (*ko_1_*).

A liner mixed effects model with just the L2 learners and the heritage speakers revealed no main effect for group but differences for sentence types. Indirect objects were rated differently from direct objects (β = 0.42, *SE* = 0.16, *t* = 2.619, *p* < 0.05) and dative subjects (β = 0.353, *SE* = 0.16, *t* = 2.154, *p* < 0.05), supporting our hypothesis. The L2 learners and the heritage speakers assigned higher acceptability ratings to ungrammatical sentences with omissions of ergative -*ne*, accusative -*ko_1_* and dative -*ko_3_* with dative experiencers than of dative -*ko_2_* with indirect objects (all comparisons significant at *p* < 0.05), as predicted.

Summarizing the findings of the AJT, we found evidence of omission of -*ne* with transitive perfective predicates and overgeneralization errors involving ergative marking with intransitive perfective predicates, although acceptability ratings for omission and overgeneralization errors were not significant for the L2 learners and the heritage speakers. The L2 learners and heritage speakers accepted errors of omission of -*ko* with human specific direct objects. Potential errors of overgeneralization of -*ko* to inanimate non-specific contexts were harder to assess because the sentences were presented in isolation, and could still receive a specific reading. The heritage speakers were more native-like in their acceptability of sentences with -*ko* as a marker of specificity with inanimate objects than the L2 learners. As for case omissions by syntactic context, indirect objects received the lowest ratings, compared to the other three conditions: ergative -*ne*, -*ko* with direct objects and -*ko* with dative experiencers.

## Discussion

The two main findings of our study were first, that morphological accuracy with Hindi case marking differed by group of bilinguals and second, that there were linguistic effects on the acquisition of the different case markers examined, modulated by semantic and syntactic complexity and input frequency.

The Hindi–English speakers from India outperformed the L2 learners of Hindi and the Hindi heritage speakers. The multilingual profile of the speakers from India is the typical reality of Hindi speakers in India, especially of the SES we tested. These speakers performed at ceiling, suggesting that knowledge of another South Asian language (some of which were not ergative) had no effect on their morphological accuracy in Hindi. By contrast, the heritage speakers and the L2 learners in our study made and accepted morphological case errors in Hindi. The three bilingual groups differed in several biographical variables, such as place of residence and upbringing, AoA of Hindi, and context of learning (naturalistic, instructed). If AoA of Hindi and context of learning were to explain the results, the speakers from India and the Hindi heritage speakers, both exposed to Hindi in a naturalistic setting early in life, would pattern together in their oral production and grammaticality judgments of Hindi case morphology. However, we found that the performance of L2 learners and the heritage speakers, who reside in the United States and were exposed to and currently use less Hindi than English and have lower proficiency in Hindi than English compared to the speakers from India (i.e., unbalanced bilinguals), was very similar. Thus, early AoA in a naturalistic setting did not matter for the acquisition of morphology. This result, to us, implies an overall dominance effect: the balanced bilinguals differed from the unbalanced bilinguals in their production and knowledge of the morphological complexity of the Hindi case system. This result is consistent with previous findings of early AoA effects for phonology but not for morphosyntactic knowledge in heritage speakers and L2 learners using production and off-line grammaticality judgment tasks (e.g., Au et al., 2002). O’Grady et al. (2001) also found no differences between L2 learners and heritage speakers of Korean on morphological case markers and relative clauses. At the same time, we note that the Hindi-dominant group and the heritage Hindi group did not only differ with regard to language dominance, but also with regard to the amount of exposure to Hindi they experience in their everyday lives. Thus, it is possible that the between-group difference that emerged in our study is actually not caused by language dominance alone, but is instead due to differences in the amount of exposure and current use of Hindi, which are experience-based components of dominance (Montrul, 2016a). Paradis (2010) study on language exposure, complexity and task type, suggests that exposure impacts the acquisition of morphology in school-age bilingual children. The children with highest exposure to English were also the most dominant in English and the ones that approached monolingual English norms more closely. Bedore et al. (2012) found that in pre-kindergarten and kindergarten-age children current language use was a better predictor of language dominance with respect to morphosyntactic measures than age of first exposure. Unfortunately, we know of no studies that tease apart exposure and language use from dominance in adults, which would be useful to establish. Still, the Hindi-dominant group, which lived in India, used Hindi more often currently than the Hindi heritage speakers. Given this state of affairs, we thus consider it more likely for now that the differences in our study are due to overall language dominance.

Amount of exposure and use of Hindi may be the reason for the morphological inaccuracies found in the two unbalanced bilingual groups. The heritage speakers in this study were exposed to Hindi naturalistically since birth for almost 20 years, but they clearly do not master case, just like the L2 learners with less than 7 years of Hindi instruction. The non-target acquisition of case manifested by the heritage speakers can be explained by reduced exposure to and use of the family language during childhood in a language-minority situation. Montrul et al. (2012) showed that adult Hindi-speaking immigrants were native-like with all these case particles, suggesting that case marking is present in the input to Hindi speakers. Therefore, it is possible that being exposed to Hindi only through the parents and using it less frequently than English may have impacted the heritage speakers’ opportunity to master case marking at native levels by adulthood. Since morphological learning depends on frequency and distribution in the input (Yang, 2002), an explanation of reduced input in childhood is compatible with the findings of the heritage speakers (O’Grady et al., 2011).

In L2 acquisition, on the other hand, non-native attainment may have two possible sources: limited exposure and restricted use of the L2 in an instructional setting (Bialystok and Hakuta, 1999), as well as the maloperation of the implicit language learning mechanisms available in childhood (DeKeyser, 2013). Our L2 learners were exposed to Hindi for 7 years at most, predominantly in an instructed setting a few hours a week. It is possible that with more input and use the L2 learners could reach the level of morphological accuracy of the Hindi speakers in India. Very advanced and near-native English-speaking L2 learners of Hindi would need to be tested to confirm this possibility. Since we did not use tests of implicit knowledge or online processing (e.g., timed grammaticality judgment tasks, self-pace reading tasks) we are unable to corroborate whether L2 learners and heritage speakers use different morphological processing mechanisms.

While finding that balanced bilinguals show better command of morphology than unbalanced bilinguals with less exposure and use of the language may be obvious, our study aimed to understand how linguistic factors may affect morphological acquisition in the weaker language. We hypothesized that the degree of accuracy on the case markers would vary as a function of syntactic and semantic complexity, and frequency in the input. The Feature Reassembly Hypothesis (Lardiere, 2009) and the Contextual Complexity Hypothesis (Hawkins and Casillas, 2008) predicted that markers that bundle more semantic and syntactic features will be more difficult to master and require more morphological computations than markers bundling fewer features (see Hawkins and Casillas, 2008 for details). This is also determined by whether or not the features and feature bundles exist in the bilinguals’ other language. Based on their different feature specifications, we expected higher accuracy with indirect objects (*ko_2_*) than with ergative -*ne*, dative experiencers (*ko_3_*) and specific direct objects (*ko_1_*). From an input-based perspective (O’Grady et al., 2011), -*ko* with indirect objects is a more stable and reliable cue than -*ko* with direct objects because *all* indirect objects are marked with -*ko* consistently whereas *some* direct objects are marked with zero. Therefore, input distribution also predicted higher accuracy on -*ko* marking of indirect object than on the other three markers.

The results of the AJT confirmed the trends observed in the oral task. The L2 learners and the heritage speakers accepted/produced more omission errors with ergative -*ne*, accusative -*ko* and dative experiencer -*ko* than with indirect objects -*ko*. There were also very few overgeneralization errors of -*ne* and -*ko*; in the case of -*ne* most overgeneralization errors were produced by the L2 learners [which suggests that they have difficulty with the application of impoverishment rule for ergativity in (10)]. Consistent with our hypothesis, the L2 learners and the heritage speakers did not omit case markers indiscriminately; variability was systematically constrained by the semantic complexity of features involved and distributional reliability of the case markers in the input.

Despite overall similar findings for the two groups, the heritage speakers exhibited more sensitivity to -*ko* use and omission than the L2 learners with inanimate direct objects, where -*ko* marking is preferred if the object is specific (DOM). Hindi does not have definite articles, and -*ko* marks definiteness and specificity. In the AJT, the heritage speakers were more accepting of -*ko* as a specificity marker with inanimate direct objects and of unmarked human non-specific objects than the L2 learners. The speakers from India and the heritage speakers produced and accepted -*ko* marking more than the L2 learners. We acknowledge that because the sentences were not presented in context, it was not possible to assess more directly whether these uses of -*ko* with inanimate non-specific objects were overgeneralization errors or renditions of specific objects, at least for the heritage speakers. These converging results from the two tasks suggest that heritage speakers seem to be more aware that -*ko* marks specificity than the L2 learners, which could be due to their longer exposure to the language since an earlier age. Further research should pursue the strength of this finding with tasks that manipulate context.

Except for the finding that heritage speakers seem to know that -*ko* marks specificity with direct objects better than the L2 learners, the reason why our heritage speakers were not more accurate on case in general may be related to the relatively advanced proficiency of the L2 learners and their exposure to reading and writing through instruction. Limited access to literacy affects heritage language development (Bayram et al., 2017). Laleko and Polinsky (2015), who investigated knowledge of topic and case markers in Korean and Japanese, found that advanced L2 learners do as well as heritage speakers recognizing different case markers in these languages. Foote (2010) also found that high proficiency L2 learners do not differ from heritage speakers of Spanish in their production of agreement.

Summarizing so far, amount of exposure to English and Hindi and patterns of language dominance are two characteristics shared by the L2 learners and the heritage speakers. Although the three groups tested were bilingual in English and Hindi, including the Hindi speakers from India, the L2 learners and the heritage speakers grew up in an English-speaking environment, were residing in an English-speaking country and were English-dominant. Non-target mastery of case morphology in the two experimental groups favors the possibility that reduced exposure to Hindi in the United States results in fewer instances of case markers in the input. The pairing of form and meanings is not always transparent (i.e., opaque) in the Hindi case system because except for indirect objects, overt case marking with ergative -*ne*, accusative -*ko* and dative -*ko* with experiencers depends on syntactic and semantic conditions (see Table 2). As a result, acquiring case marking in Hindi requires sustained frequent input to learn the form-meaning mappings, which is what heritage speakers typically lack.

Finally, the dominant language itself may contribute to the morphological patterns found in the two English-dominant groups. Because English is not an ergative language and does not mark case overtly, it may also have contributed to difficulty marking case consistently in Hindi, the weaker language. On Keine’s (2007) analysis of ergative -*ne* and accusative -*ko*, overt marking is the default and zero marking is the result of impoverishment rules, which predicts more errors of overgeneralization of -*ne* and -*ko* than omission. However, when the heritage speakers made errors, in general there were more omission than overgeneralization in the production task and no difference in the acceptability judgments between the two ungrammatical sentence types in the AJT. The L2 learners made both omission and overgeneralization errors, especially more overgeneralization errors with -ne in the oral production task, suggesting that they have yet fully acquired the impoverishment rule in (10). Perhaps the difference error patterns with ergatives may be related to the fact that L2 learners were receiving instruction and were more aware of ergativity marking than the heritage speakers, who were not receiving Hindi instruction. As for omission errors, or both L2 learners and heritage speakers, unmarked case in English is a very likely source of language influence and case omissions in Hindi. With the ergative, the heritage speakers and L2 learners may be reinforcing the nominative-accusative pattern from English in Hindi. A similar explanation for errors with ergative marking in minority language bilinguals has been advanced for Dyirbal in Australia (Schmidt, 1985) and Basque in Spain (Austin, 2007), where the ergative language is in contact with a nominative-accusative language. When -*ne* is present in Hindi, object agreement is with the object, not with the subject. If the English-dominant bilinguals were assuming a nominative-accusative pattern for the two languages they would consistently produce agreement with the subject. We examined the production data and found that some heritage speakers omitted -*ne* and produced subject agreement but others produced object agreement. We also found ungrammatical cases of -*ne* omission and default agreement. In general, we did not observe any distinct pattern to support a change from ergative-absolutive to nominative-accusative. Our AJT did not include sentences testing ergativity marking with imperfective predicates and sentences manipulating different types of subject, object and default agreement errors. We acknowledge that this limitation prevents us from evaluating more directly transfer of the nominative-accusative system of English to the split ergative system of Hindi. This question requires a more in depth experimental study of ergativity and agreement patterns in Hindi as a heritage language and as a second language.

Finally, English dominance may also explain the omission of accusative -*ko* with animate specific direct objects as well as the omission of -*ko* with dative subjects, since English does not presently mark case overtly with direct objects or experiencer subjects. Spanish is a nominative-accusative language but like Hindi, it also marks animate specific indirect objects overtly. Studies on L2 Spanish (Guijarro Fuentes, 2012) and Spanish as a heritage language by speakers of English (Montrul, 1998, 2016b) have found high rates of case omission of the preposition “a” with animate objects and dative experiencer subjects. Therefore, the morphological variability found in the present study can easily be explained by dominant language transfer, in this case English, in the bilinguals with Hindi as weaker language.

## Conclusion

Our results confirmed that bilinguals with Hindi as non-dominant language show morphological variability and instability with morphological case, unlike fluent Hindi speakers who are balanced bilinguals. Case markers linked to semantic features like perfectivity of verbs, and animacy or specificity of nouns (ergative -*ne* and some instances of -*ko*) were more prone to omission than case markers that are more predictable in the input, like the dative case of indirect objects. For both the L2 learners and the heritage speakers, the quantity and complexity of features bundled in morphemes coupled with amount of input and use of Hindi (reflected in overall proficiency) affect the strength of form-meaning mappings and their mastery of the case morphology. Morphological variability may be reinforced by knowledge of English, the dominant language in L2 learners and heritage speakers, which does not mark these cases overtly. AoA of Hindi may explain the heritage speakers’ superior sensitivity to -*ko* as a specificity marker, but it did not modulate the overall level of case accuracy in this study. The acquisition and mastery of morphology in bilinguals seems to be determined more by amount of input and use than by age of onset of bilingualism and length of language use, especially in unbalanced bilinguals.

## Ethics Statement

This study was carried out in accordance with the recommendations of the Institutional Review Board at the University of Illinois. The protocol was approved by the Office for the Protection of Human Subjects at the University of Illinois. All subjects gave written informed consent in accordance with the Declaration of Helsinki.

## Author Contributions

SM designed the overall project, performed statistical analyses, and wrote the article. AB, RB, and VP created the Hindi version of the tests. AB and VP tested participants, transcribed, and coded oral data. AB and RB contributed to the writing as well.

## Conflict of Interest Statement

The authors declare that the research was conducted in the absence of any commercial or financial relationships that could be construed as a potential conflict of interest.
